# On the Catalase Activity of the Liver Soluble Proteins in Rats Fed on 4-Dimethylaminoazobenzene and Localization of the Protein-Bound Dyes

**DOI:** 10.1038/bjc.1955.71

**Published:** 1955-12

**Authors:** F. Abrignani, V. Mutolo


### ON THE CATALASE ACTIVITY OF THE LIVER SOLUBLE PROTEINS IN RATS FED ON 4-DIMETHYLAMINOAZOBENZENE AND LOCALIZATION OF THE PROTEIN-BOUND DYES.

F. ABRIGNANI AND V. MUTOLO.

*From the Biological Department, Centro Tumori, Palermo.*

Received for publication October 3, 1955.

AMONG all the tissues of the tumour-bearing animal the liver appears to be the most sensitive to the presence of the tumour. Properly it is in the field of enzymology that this sensitivity attains its clearest expression by the lowering of the liver catalase activity. This interesting phenomenon arises systematically with all spontaneous, induced or transplantable tumours in rats and mice of various strains (Colwell, 1910 ; Rosenthal, 1912 ; Magat, 1924 ; Greenstein, 1943, 1954) and also in frogs (Lucké and Berwick, 1954) and chickens (Adams, 1954). Hepatomas, lymphomas, sarcomas, carcinomas, etc., all exert a similar depressing effect on the liver catalase activity as if the tumours, regardless of etiology or histogenesis, converge towards a common type of tissue possessing an identical property.

Now if we consider that of all the individual enzyme systems examined in the tumour-bearing host, only the hepatic catalase is firmly and markedly affected, and that in other conditions, in pregnancy and after subcutaneous embryonic implant, there is no change in the normal level of the liver catalase, we can reach the suggestive conclusion that the depression of the catalase is an exclusive expression of the cancerous affection. This conclusion is also in accordance with the reversibility of the enzymatic activity after the complete removal of the tumour.

But the possibility of applying this finding to early diagnosis of human cancer has been unsuccessful, and Young, Collier and Homburger (1947), and Mutolo and Abrignani (1954) found no difference in catalase activity between men suffering from cancer and those in normal health.

To explain the decrease of the catalase activity Weil-Malherbe and Schade (1948) hypothesized that this phenomenon might be caused by the reduction of the catalase synthesis following the high protein requirements of the tumour ; however the liver catalase of the tumour-bearing animals, regardless of the nature of the diet, was constantly lowered.

The eventuality of the humoral transmission of some anticatalase agent from the tumour has been examined by Lucké, Berwick and Zecker (1952) who showed that in parabiotic rats the level of the liver catalase activity of the non-tumour-bearing partner is reduced. Furthermore Nakahara and Fukuoka (1950) succeeded in isolating from a number of human tumours a protein-like toxic substance (toxohormone) which, when injected into mice, brings about a lowering in the liver catalase activity. Finally Hargreaves and Deutsch (1952) found that boiled extracts of tumours could inhibit *in vitro* the activity of crystalline catalase.

Starting with this observation we have discovered that the ovomucoid acquires, after boiling, a strong inhibitory power against catalase and we have supposed that probably the anticatalase activity of the tumours may be due to a glycoprotein (Abrignani and Mutolo, 1954).

The mechanism of the fall in the liver catalase activity in tumour-bearing animals and why this effect begins to be readily measurable only at a stage when the tumour is at least 5 per cent of the body weight of the host is not yet understood. It is possible that the toxic substance elaborated in relatively insufficient amounts by a small size tumour does not affect the liver catalase since probably it is neutralized in the blood stream before entering the liver. Hence the lowering of the hepatic catalase activity should be observable not only when the small tumour itself grows in the liver but also in the earliest steps of the neoplastic induction.

In an attempt to prove the above-mentioned hypothesis we have studied the behaviour of the catalase activity during the first steps of the liver carcinogenesis in rats fed on 4-dimethylaminoazobenzene (DAB).

In regard to this Miller and Miller (1947, 1953), have demonstrated that protein-bound dyes appear in the liver shortly after the feeding of DAB and continue to accumulate until a maximum level is reached at about one month ; thereafter the level decreases progressively (Miller and Miller, 1952). In contrast, these protein-bound dyes are absent in the fully developed liver tumours (Miller and Miller, 1947 ; Price, Miller, Miller and Weber, 1949).

The studies on the metabolism of this dye have shown that the major share of of the dye is metabolized via reductive cleavage of the azo-linkage, hydroxylation of the ring in the 4′-position, and oxidative removal of the N-methyl groups. Only a small percentage of a dye derivative becomes chemically bound to certain of the liver proteins (McDonald, Plescia, Miller and Miller, 1953 ; Stevenson, Dobriner and Rhoads, 1942). These protein-bound dyes are highly specific for the induction of the hepatoma in the rat liver, since they cannot be detected in the liver of the animals which are not susceptible to the carcinogenic action of DAB (Miller and Miller, 1952).

The riboflavin, added to the diet, was discovered to be one of the most potent inhibitors of the induction of the tumours by DAB (Kensler, Sugiura, Young, Halter and Rhoads, 1941).

Furthermore it has been observed that the major portion of the protein-bound amino azo dye derivatives is contained in the slower sedimenting class of soluble, non-particulate proteins of rat liver (Sorof, Cohen, Miller and Miller, 1951) and on electrophoretic analysis the protein-bound dyes were found approximately to relate to the slowly moving *h* component (Sorof, Golder and Ott, 1954).

The present paper deals with the catalase activity of the liver soluble, non-particulate, proteins of rats feeding for a week on DAB. For the purpose of this research the isolation and the separation of the liver soluble proteins have been performed by salting-out method which has allowed us to localize both the fraction containing the protein-bound dyes and that possessing the catalase activity.

### METHODS.

*Care of animals.*—In all, 40 albino rats young and adult of both sexes weighing 110 to 160 g. were used. Two rats at a time were fed on polished boiled rice, carrot, cod liver oil and water *ad libitum.* After they were accustomed to this diet, one rat was given daily for a week 30 mg. of 4-dimethylaminoazobenzene dissolved by heat in the cod liver oil. The other rat, used as control, was fed continuously on the rice-carrot diet without azo dye. Consecutively the rest of the animals, in couples, were fed as above. All the animals in the experiment were housed at 23° C. separately in screen-bottomed cages. The mortality of the animals treated with DAB was 40 per cent.

*Isolation of the soluble proteins of rat liver.*—The rats, after fasting for 16–20 hours, were killed with ether and in a cold room (0–5° C.) the livers were perfused for 10 minutes *in situ* with cold 0·15M NaCl through the vena porta. The livers were removed and a small fragment was taken away to ascertain qualitatively the approximate level of protein-bound dye in the liver as described below. Then the livers were quickly weighed and placed in a freezer at − 10° C. for 12–16 hours. After gradual thawing the liver was homogenized at 0° C. with a glass homogenizer with teflon plunger in 2 volumes of cold solution of 0·1M LiCl–0·04M $NaHCO_3$–0·01M $Na_2CO_3$. After 10 minutes it was centrifuged at 24,000 × g. for 30 minutes at 0° C. The sediment was extracted again for 10 minutes in 1 volume of the same extracting solution and was centrifuged as above. The supernatants were mixed and centrifuged at 0° C. at 24,000 × g. for 60 minutes. The final supernatants (total extracts) were clear amber from the control animals and brown from the animals fed on DAB.

*Separation of the total extract of liver soluble proteins.*—The total extract fractions were separated by means of salting-out using ammonium sulphate to 0·50 and 0·65 of saturation. The salting-out procedure was accomplished by dialysis at 5° C. for 24 hours against 10 volumes of the solution of ammonium sulphate buffered with phosphate at pH 7·3 replaced twice. The precipitates of the fractions at 0·50 and 0·65 of saturation were brought to the original volumes with cold solution of 0·15M NaCl–0·1M $NaHCO_3$.

*Bound dye analysis.*—In the liver the bound dye was revealed by fixing one millimetre thick cross sections of liver in a mixture of formalin, acetic acid, ethanol, and water in the proportion of 1 : 1 : 3 : 5 respectively. After 5 minutes the hardened and bleached sections were blotted and transferred to 10 per cent trichloroacetic acid. Five minutes after the sections ceased to float, they generally had assumed a maximum pink coloration due to the protein-bound dye (Miller, Miller, Sapp and Weber, 1949).

In total extracts, in fractions 0·50 and 0·65 of saturation, and in the final supernatants, the bound dye was analysed by adding to their aliquot an equal volume of 20 per cent trichloroacetic acid and then, after stirring, the mixture was centrifuged at 5000 r.p.m. for 10 minutes. The sediments which showed a characteristic pink coloration were those endowed with the protein-bound dyes.

*Determination of catalase activity.*—Catalase activity in total extracts, fractions, and final supernatants of the liver soluble proteins was determined after dialysis at 5° C. for 72 hours against 50 volumes of 0·15M NaCl–0·1M $NaHCO_3$ replaced every 12 hours and then centrifuged at 5000 r.p.m. for 10 minutes to remove the slight precipitate present. 1·5 ml. of the perfectly clear samples taken in examination, containing 100 $\mu$g. total N, was kept at 0° C. for 10 minutes then 5 ml. of 0·025M hydrogen peroxide buffered with phosphate at pH 7·4 was added. After 2–5 minutes at 0° C. the enzymatic reaction was stopped with 0·6 ml. of 9·37M sulphuric acid. Successively at room temperature 3 ml. of 2·4M trichloroacetic acid were added, shaking well. The solution was left to stand for 15 minutes and filtered. To 5 ml. of the clear filtrate 1 ml. of 0·903M potassium iodide was added.

After 15 minutes, the colour developed was measured at a wave length of 560 m$\mu$. with a Beckman spectrophotometer and the colorimetric readings were related to a standard curve of hydrogen peroxide. The catalase activity was estimated from the amount of split of the hydrogen peroxide and was calculated subtracting the value of the tests from that of the hydrogen peroxide control.

*N and P analyses.*—The total N was assayed by direct Nesslerization and total P following the Allen's method (1940). The total extract, the fractions, and the final supernatants were previously dialyzed as described above in determination of catalase activity.

*Symbols.*—Total extract, fractions, and final supernatant of the liver soluble proteins from the DAB treated and control rats were abbreviated to te-DAB ; 0·50-DAB ; 0·65-DAB ; s-DAB and te-C ; 0·50-C ; 0·65-C ; s-C respectively.

### RESULTS.

*N and P content of the liver soluble proteins and their fractions.*—Table I summarizes the analytical data for the content of N and P of the te-DAB ; 0·50-DAB ; 0·65-DAB ; s-DAB compared to the te-C ; 0·50-C ; 0·65-C ; s-C.

TABLE I.—*$\mu$g. of N and of P/1000 $\mu$g. total N in the Different Fractions of the Soluble Proteins of the Liver in the DAB and Control Animals.**

| | N. | P. | N/P. |
|---|---|---|---|
| te-DAB | 1000·0 | 30·60±1·24 | 32·70 |
| 0·50-DAB | 490·0±7·57 | 10·70±0·57 | 45·70 |
| 0·65-DAB | 53·7±1·36 | 0·75±0·67 | 71·70 |
| s-DAB | 112·0±1·47 | 1·94±0·73 | 57·70 |
| te-C | 1000·0 | 31·90±1·46 | 31·40 |
| 0·50-C | 540·0±4·04 | 10·10±0·96 | 54·00 |
| 0·65-C | 132·5±1·44 | 1·29±0·36 | 102·00 |
| s-C | 89·6±0·92 | 1·04±0·92 | 86·00 |

* Average values of 12 experiments.

te-DAB = Total extract of the liver soluble proteins from the rats fed on DAB.
te-C = ,, ,, ,, ,, ,, ,, ,, ,, control rats.
0·50-DAB = Fraction at 0·50 of saturation of the total extract from the rats fed on DAB.
0·50-C = ,, ,, 0·50 ,, ,, ,, ,, ,, ,, ,, control rats.
0·65-DAB = ,, ,, 0·65 ,, ,, ,, ,, ,, ,, ,, rats fed on DAB.
0·65-C = ,, ,, 0·65 ,, ,, ,, ,, ,, ,, ,, control rats.
s-DAB = Final supernatant of the total extract from the rats fed on DAB.
s-C = ,, ,, ,, ,, ,, ,, ,, control rats.

The data in Table I show that the amount of N recovered in the 0·50 and 0·65 fractions is lower in the DAB animals than in the control animals. This is especially relevant from the 0·65 fraction. On the contrary, the supernatant, which evidently consists of rather low molecular proteins, is considerably larger in the DAB animals. Also the P content indicates a shift from the 0·65 to the supernatant, although this is smaller than the corresponding shift of N. That indicates a considerable breaking-down of the proteins especially of the 0·65 fraction in the liver of the DAB treated animals.

*Localization of the protein-bound dyes.*

Using the salting-out procedure with ammonium sulphate, the protein-bound dyes in the liver soluble proteins of rats fed on DAB are localized in the fraction precipitating at 0·65 of saturation. This is revealed by the pink colour that this fraction presents to the bound dye analysis. However, sometimes a very few protein-bound dyes can be found also in s-DAB. This is a further indication of the break-down of the proteins of this fraction to compounds of lower molecular weights. The 0·50-DAB never shows protein-bound dye.

*Behaviour of the catalase activity.*

In examination of catalase activity in the total extracts of liver soluble proteins, we have found that the te-DAB exhibits a lowering of the enzymatic activity as expressed by the split of hydrogen peroxide in 2 minutes of reaction at 0° C. Indeed the te-DAB gives values of catalase activity ranging between 625 $\mu$g. and 925 $\mu$g./100 $\mu$g. N of hydrogen peroxide break-down against 1300–1450 $\mu$g./100 $\mu$g. N of the te-C. The lowering of catalase activity of the te-DAB is 55·3 per cent. In experiments on fractions of both the DAB and control animals only the 0·50 fraction exhibits catalase activity which, however, is lower than that of the original total extract. Therefore it was estimated after 5 minutes of reaction instead after 2 minutes. This decrease of the activity can be ascribed to the adsorption of the catalase on the cellophane tubing during dialysis (Hennichs, 1926 ; Sumner, 1941). The 0·50-DAB fraction shows the same lowering of the catalase activity with respect to the corresponding fraction of the control animals just as it has been observed in the total extract. In fact, the activity of this fraction gives values 400–500 $\mu$g. in respect to 825–1250 $\mu$g. of the 0·50-C. The reduction in catalase activity of the 0·50-DAB is 43·9 per cent. Furthermore the fraction at 0·65 of

TABLE II.—*Catalase Activity in the Total Extracts and Fractions of the Liver Soluble Proteins in the DAB and Control Animals.**

| Exper. No. | te-C†. | te-DAB†. | 0·50-C‡. | 0·50-DAB‡. | 0·65-C‡. | 0·65-DAB‡. | s-C‡. | s-DAB‡. |
|---|---|---|---|---|---|---|---|---|
| 1 | 1400 | 900 (64·3) | 825 | 525 (63·5) | 105 | 125 | 0 | 0 |
| 2 | 1350 | 625 (46·3) | 1250 | 540 (35·2) | 80 | 115 | 0 | 0 |
| 3 | 1425 | 700 (49·1) | 1050 | 430 (40·9) | 105 | 120 | 0 | 0 |
| 4 | 1300 | 720 (55·4) | 1125 | 465 (41·3) | 95 | 125 | 0 | 0 |
| 5 | 1325 | 815 (61·5) | 980 | 510 (52·0) | 120 | 135 | 0 | 0 |
| 6 | 1450 | 925 (63·8) | 1100 | 400 (36·3) | 125 | 125 | 0 | 0 |
| 7 | 1410 | 820 (53·1) | 1150 | 425 (36·9) | 100 | 130 | 0 | 0 |
| 8 | 1315 | 780 (59·3) | 975 | 530 (34·4) | 115 | 135 | 0 | 0 |
| 9 | 1425 | 675 (47·4) | 1065 | 525 (49·3) | 85 | 125 | 0 | 0 |
| 10 | 1350 | 690 (51·1) | 1210 | 410 (33·9) | 80 | 120 | 0 | 0 |
| 11 | 1325 | 740 (55·8) | 1210 | 540 (44·6) | 100 | 115 | 0 | 0 |
| 12 | 1300 | 750 (57·7) | 1115 | 435 (39·0) | 90 | 130 | 0 | 0 |
| Average | 1364 | 761 (55·3) | 1087 | 469 (43·9) | 100 | 125 | 0 | 0 |

* In terms of $\mu$g. of hydrogen peroxide split at 0° C. by total extract and fraction containing 100 $\mu$g. total N.
† After 2 minutes' reaction.
‡ After 5 minutes' reaction.
The percentage diminution of the catalase activity is expressed in parentheses.
te-C = Total extract of the liver soluble protein from the control rats.
te-DAB = ,, ,, ,, ,, ,, ,, ,, ,, rats fed on DAB.
0·50-C = Fraction at 0·50 of saturation of the total extract from control rats.
0·50-DAB = ,, ,, 0·50 ,, ,, ,, ,, ,, ,, the rats fed on DAB.
0·65-C = ,, ,, 0·65 ,, ,, ,, ,, ,, ,, the control rats.
0·65-DAB = ,, ,, 0·65 ,, ,, ,, ,, ,, ,, the rats fed on DAB.
s-C = Final supernatant of the total extract from the control rats.
s-DAB = ,, ,, ,, ,, ,, ,, ,, rats fed on DAB.

saturation exhibits a very small catalase activity possibly due to the enzyme contamination. The catalase activity observed in these fractions is too weak for a comparison to be made between DAB and control animals. We never have found catalase activity in the s-DAB and s-C. The values of the catalase activity of the total extracts and fractions are shown in Table II.

### DISCUSSION.

To study the behaviour of the catalase activity in the first steps of the tumoural induction our animals have been given a higher amount of 4-dimethylaminoazobenzene (30 mg. daily) than usually employed. As is well known, the level of hepatic bound dye increases with increasing the dietary level of dye. In this way the protein-bound dye is easier to detect after a week, and at the same time the action of the carcinogenic agent is more powerful. The results we have obtained with the soluble proteins seem to demonstrate that the lowering of the catalase activity can be disclosed in the earliest stages during the induction of liver tumours only.

The catalase activity in rats fed on DAB has been investigated by Euler, Hasselquist and Eriksson (1952) and by Mori and Momoki (1952). They describe a decrease of catalase activity in the liver tissue of rats fed on 0·06 per cent DAB in the diet for a long period of time, whereas the activity of kidney catalase did not change. Our results agree with these findings. However, with our method of extraction and fractionation of the liver soluble proteins, we have succeeded in showing that the fraction to which the dye is bound is different from the one bearing catalase activity. Thus it appears likely that the amino azo dye does not act directly on the liver catalase and hence the decrease of its activity is probably related to some inhibitor called forth by the metabolic changes produced in liver by DAB. In this view the lowering of the catalase activity which we have observed in the first steps of the neoplastic induction may be regarded as similar to that occurring in large tumours.

Furthermore, the liver protein fractions of rats fed on DAB show considerable differences in N and P content and in N/P ratio compared to the control rats. The N and P content in fractions at 0·65 of saturation, where the protein-bound dyes are localized, is decreased in respect to that of the non-treated animals. The level of N and P is always more elevated in the supernatant of the treated animals. The N/P ratio of all the fractions and supernatant from rats fed on DAB presents a constant lowering in respect to the control animals.

These results suggest that the liver soluble proteins from rats treated with DAB appear as if they were broken down. In this way the lower precipitation of the soluble proteins at 0·50 and 0·65 of saturation and the larger amount of the non-precipitable proteins in the supernatant can be explained.

### SUMMARY.

1. The liver of rats both not fed on and fed on 30 mg. of 4-dimethylaminoazobenzene daily for one week were perfused, homogenized and the soluble, non-particulate proteins extracted. The total extracts were separated by salting-out, using ammonium sulphate at 0·50 and 0·65 of saturation. The total extract, the fractions at 0·50 and 0·65 of saturation, and the final supernatant were analysed for N and P content, protein-bound amino azo dye, and catalase activity.

2. Using the salting-out procedure the major part of the protein-bound dyes were localised in the fraction precipitated at 0·65 of saturation, whereas the fraction at 0·50 was endowed with the total catalase activity.

3. In rats treated with DAB the total extracts of the liver soluble proteins show a distinct lowering of the catalase activity, this lowering is also observed in fraction at 0·50 of saturation where all the original catalase is localized.

4. The content of N and P of the total extract and 0·50 fraction from rats fed on DAB was comparable to those of control rats. The fraction at 0·65 of saturation in which occur the protein-bound dye but not the catalase, presents a considerable decline of N and P in animals fed on DAB in respect to the controls. On the contrary the amount of N and P of the final supernatant is higher in DAB treated rats than in controls. These changes of the N and P content were regarded as expression of the metabolic alteration caused by the carcinogen on the liver.

5. The lowering of the catalase activity seemed to refer to an anticatalase substance, brought about by the metabolic changes which occur in the liver during the neoplastic induction more than to a direct action of the azo dye upon the catalase, since the catalase and the aminoazo dye were localized in different fractions. Perhaps the phenomenon of the fall of the liver catalase activity in tumour-bearing animals arises already in the first steps of the neoplastic induction, but it cannot be early detected in tumours far from the liver because the tumoural anticatalase substance is neutralized in the blood stream or in some tissues before reaching the liver.

We are indebted to Prof. A. Monroy, Institute of Comparative Anatomy, Palermo, for the critical discussion of the experimental results.

This Research was supported by a grant from the Lega Italiana per la lotta contro i Tumori, and was subject of a previous report to the meeting of the Società Italiana di Biologia Sperimentale, section of Palermo, April 22, 1955.

### REFERENCES.

ABRIGNANI, F. AND MUTOLO, V.—(1954) *Experientia*, **10**, 470.
ADAMS, D. H.—(1954) *Brit. J. Cancer*, 7, 501.
ALLEN, R. J. L.—(1940) *Biochem. J.*, **34**, 858.
COLWELL, A.—(1910) *Arch. Middx. Hosp.*, **19**, 64.
EULER, H., VON, HASSELQUIST, H. AND ERIKSSON, E.—(1952) *Arkiv. Kemi*, **4**, 487.
GREENSTEIN, J. P.—(1943) *J. nat. Cancer Inst.*, **3**, 397.—(1954) 'Biochemistry of Cancer.' New York (Academic Press).
HARGREAVES, A. B. AND DEUTSCH, H. F.—(1952) *Cancer Res.*, **12**, 720.
HENNICHS, S.—(1926) *Biochem. Z.*, **171**, 314.
KENSLER, C. J., SUGIURA, K., YOUNG, N. F., HALTER, C. R. AND RHOADS, C. P.—(1941) *Science*, **93**, 308.
LUCKÉ, B. AND BERWICK, M.—(1954) *Proc. Amer. Ass. Cancer Res.*, **1**, 30.
*Iidem* AND ZECKER, I.—(1952) *J. nat. Cancer Inst.*, **13**, 581.
MAGAT, J.—(1924) *Z. ges. exp. Med.*, **42**, 95.
McDONALD, J. C., PLESCIA, A. M., MILLER, E. C. AND MILLER, J. A.—(1953) *Cancer Res.*, **13**, 292.
MILLER, E. C. AND MILLER, J. A.—(1947) *Ibid.*, **7**, 468.—(1952) *Ibid.*, **12**, 547.—(1953) *Advances in Cancer Res.*, **1**, 339.
*Iidem*, SAPP, R. W. AND WEBER, G. M.—(1949) *Cancer Res.*, **9**, 336.
MORI, K. AND MOMOKI, S.—(1952) *Gann.*, **43**, 431.

MUTOLO, V. AND ABRIGNANI, F.—(1954) *Boll. Soc. ital. Biol. sper.*, **30**, 209.
NAKAHARA, W. AND FUKUOKA, F.—(1950) *Gann.*, **41**, 47.
PRICE, J. M., MILLER, J. A., MILLER, E. C. AND WEBER, G. M.—(1949) *Cancer Res.*, **9**, 96.—(1949) *Ibid.*, **9**, 398.
ROSENTHAL, E.—(1912) *Dtsch. med. Wschr.*, **38**, 2270.
SOROF, S, COHEN, P. P., MILLER, E. C. AND MILLER, J. A.—(1951) *Cancer Res.*, **11**, 383.
*Idem*, GOLDER, R. H. AND OTT, M. G.—(1954) *Ibid.*, **14**, 190.
STEVENSON, E. S., DOBRINER, K. AND RHOADS, C. P.—(1942) *Ibid.*, **2**, 160.
SUMNER, J. B.—(1941) *Advanc. Enzymol.*, **1**, 163.
YOUNG, N. F., COLLIER, V. AND HOMBURGER, F.—(1947) *Proc. Soc. exp. Biol., N.Y.*, **66**, 323.
WEIL-MALHERBE, H. AND SCHADE, R.—(1948) *Biochem. J.*, **43**, 118.


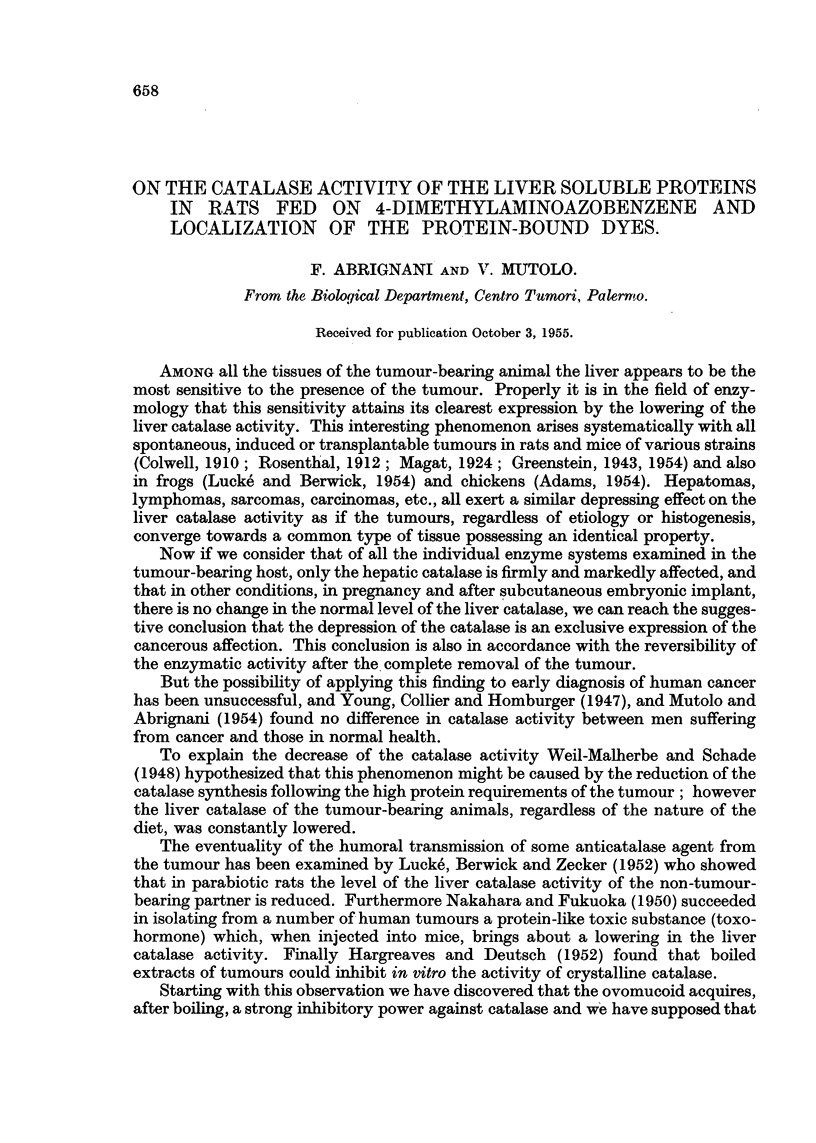

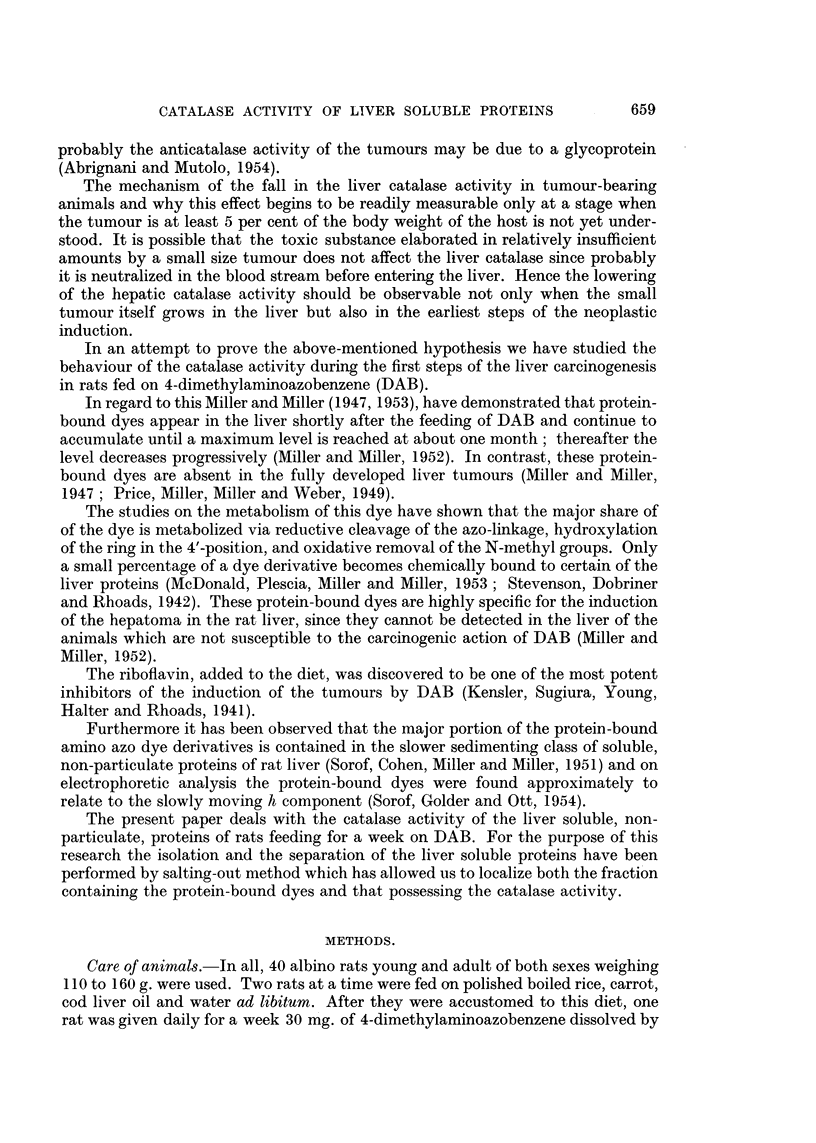

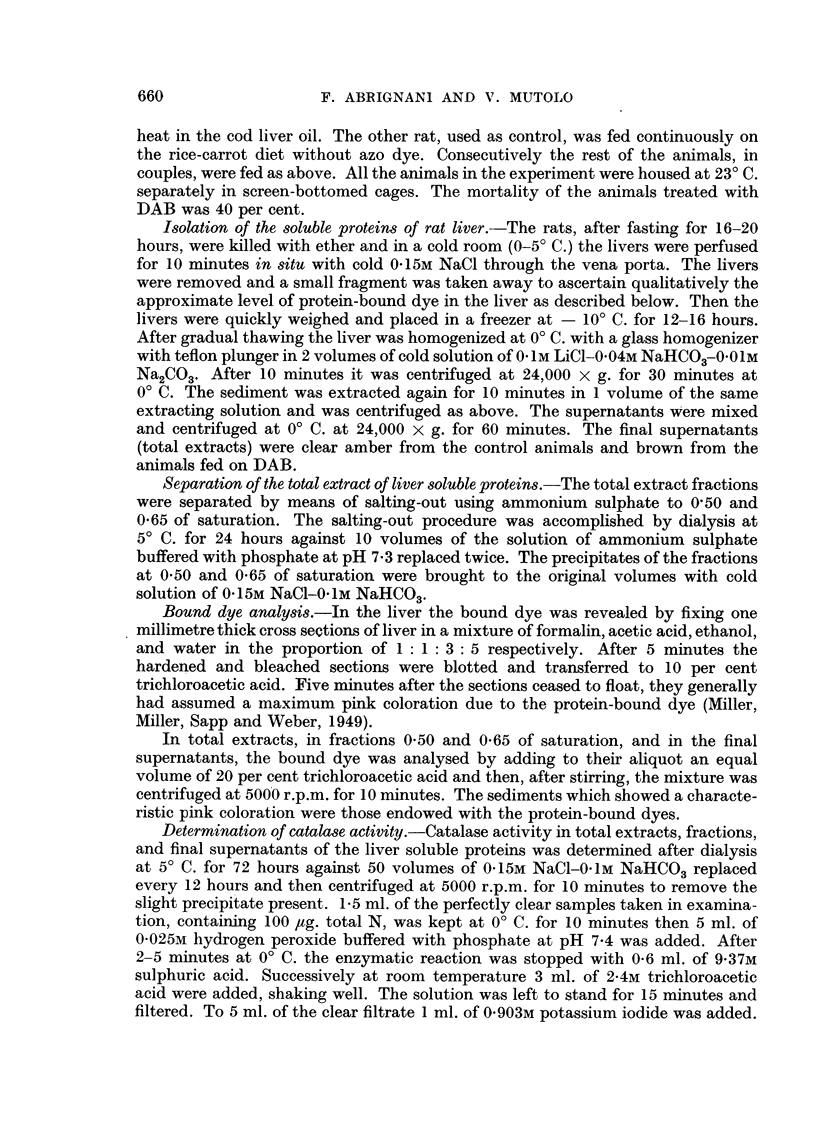

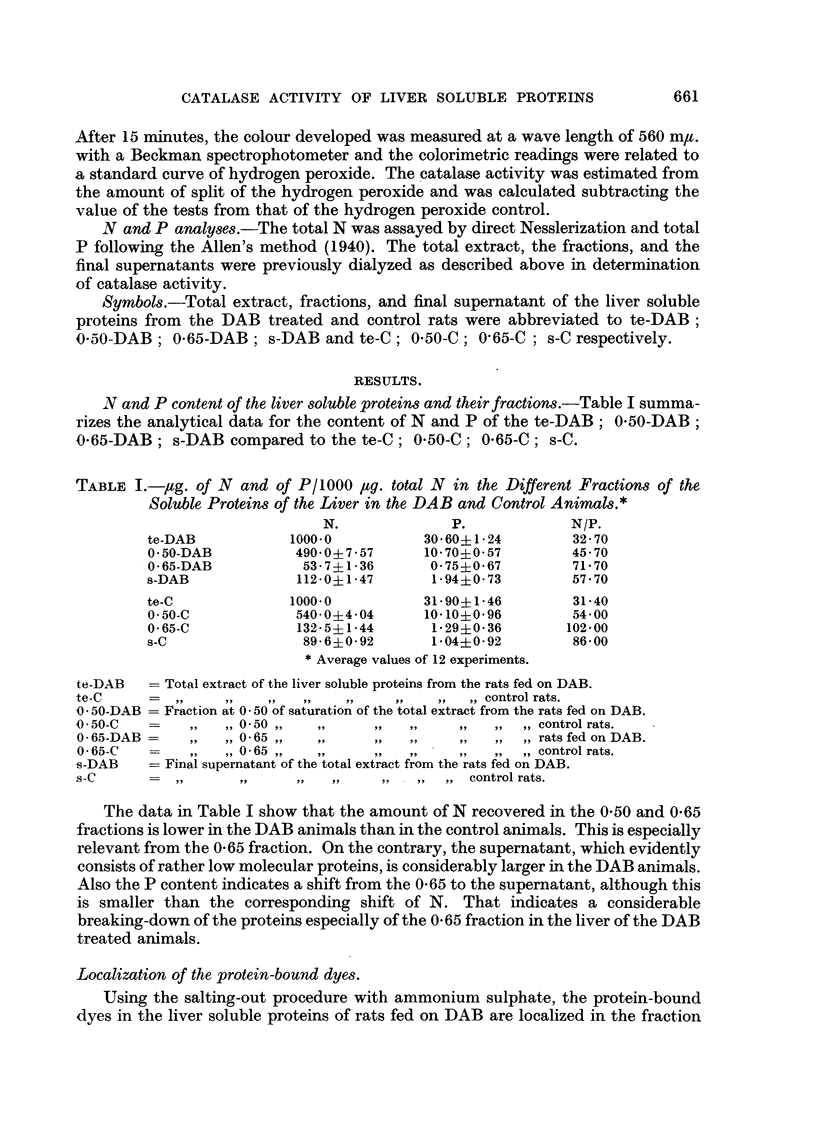

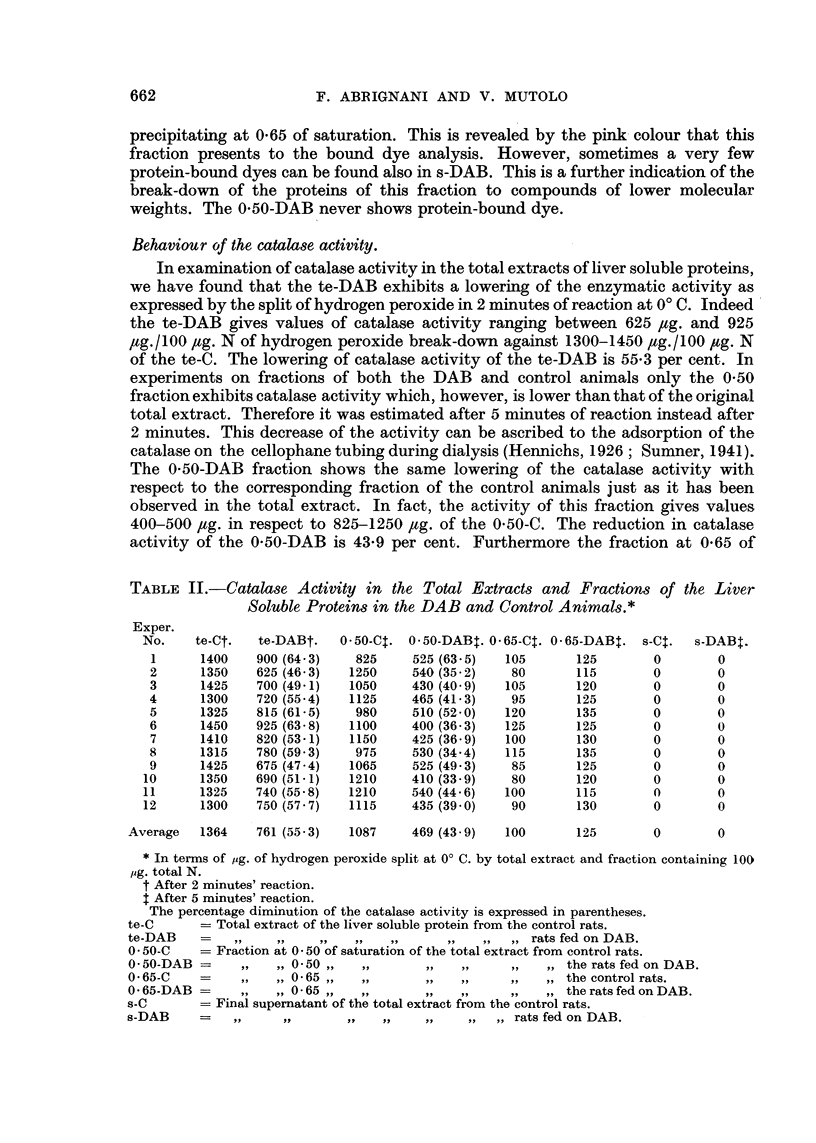

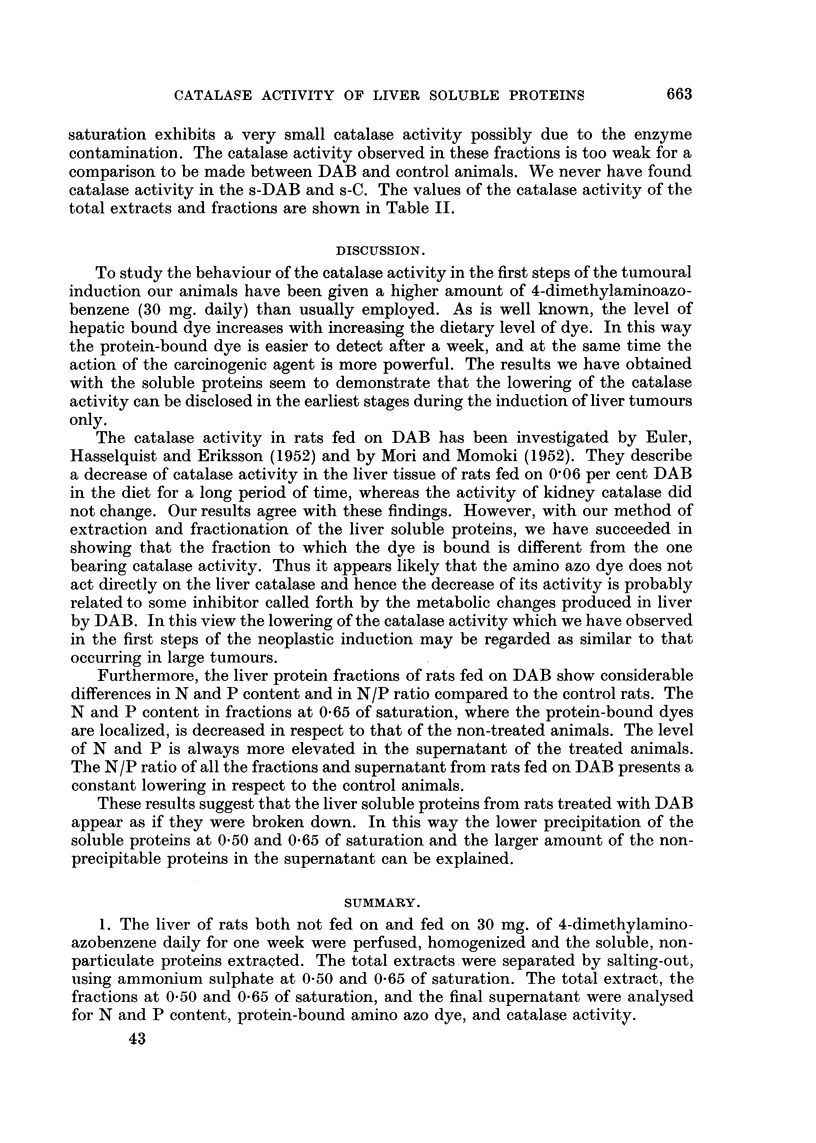

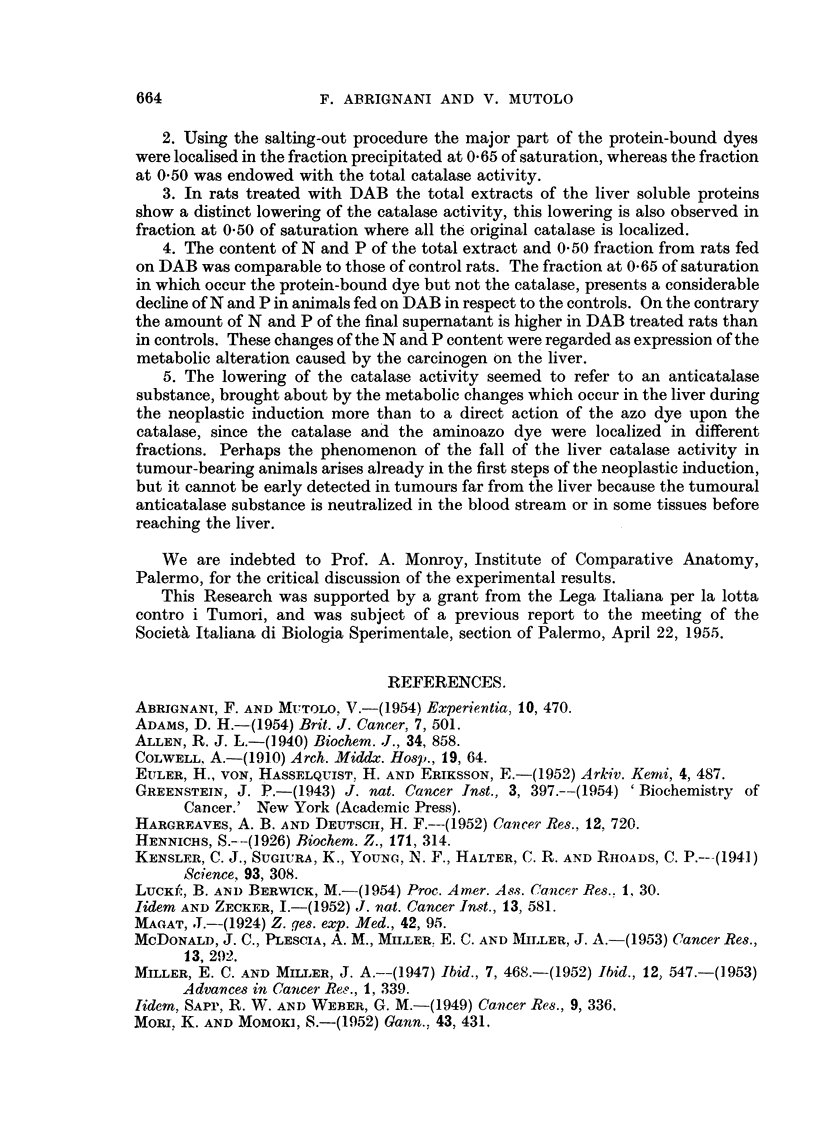

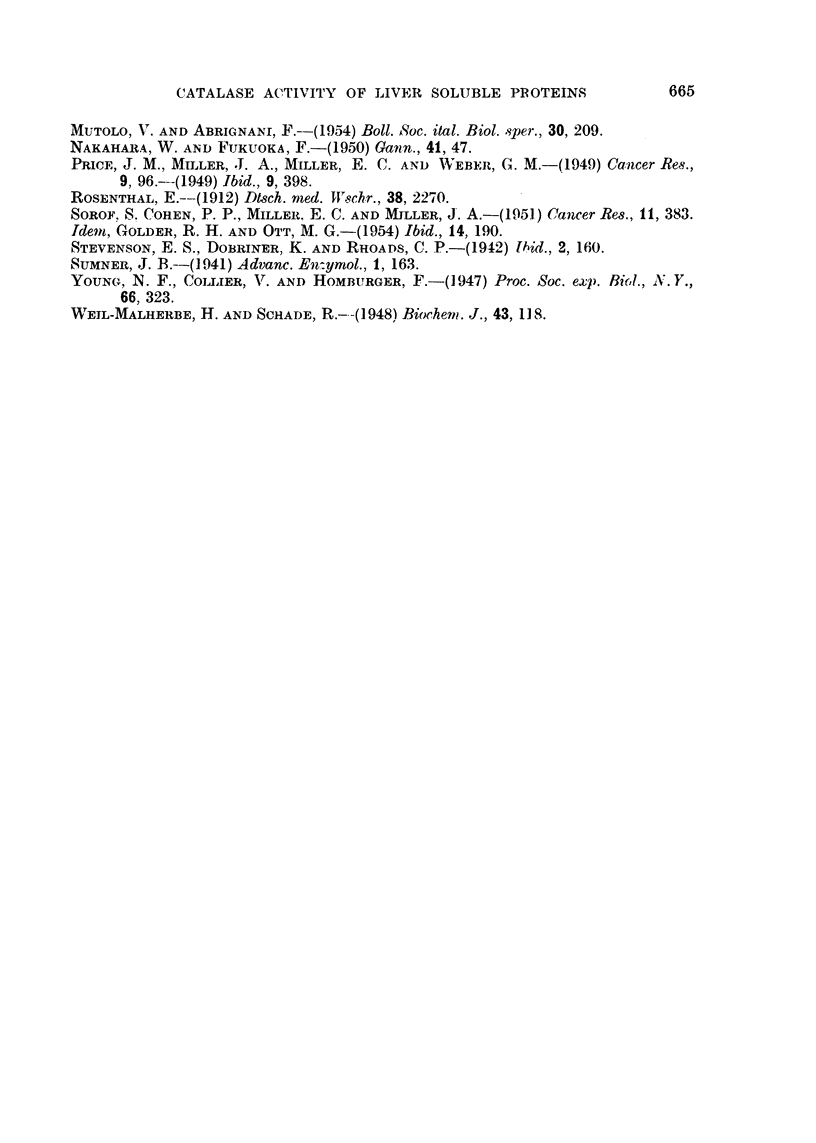


## References

[OCR_00416] ABRIGNANI F., MUTOLO V. (1954). In vitro inhibition of catalase by ovomucoid.. Experientia.

[OCR_00419] Allen R. J. (1940). The estimation of phosphorus.. Biochem J.

[OCR_00430] Kensler C. J., Sugiura K., Young N. F., Halter C. R., Rhoads C. P. (1941). PARTIAL PROTECTION OF RATS BY RIBOFLAVIN WITH CASEIN AGAINST LIVER CANCER CAUSED BY DIMETHYL-AMINOAZOBENZENE.. Science.

[OCR_00438] MACDONALD J. C., PLESCIA A. M., MILLER E. C., MILLER J. A. (1953). The metabolism of methylated aminoazo dyes. III. The demethylation of various N-methyl-C14-aminoazo dyes in vivo.. Cancer Res.

[OCR_00452] MUTOLO V., ABRIGNANI F. (1954). Attività della catalasi epatica nell'uomo affetto da cancro.. Boll Soc Ital Biol Sper.

[OCR_00460] SOROF S., COHEN P. P., MILLER E. C., MILLER J. A. (1951). Electrophoretic studies on the soluble proteins from livers of rats fed aminoazo dyes.. Cancer Res.

